# Child's Temperament as Risk Factor for Preoperative Anxiety—A Secondary Analysis of the ALPAKA Trial

**DOI:** 10.1002/pan.70150

**Published:** 2026-02-18

**Authors:** Thorben Jacobi, Sebastian Walter, Andrea Pickartz, Georg Baller, Tobias Becher, Ingmar Lautenschläger, Armin Sablewski

**Affiliations:** ^1^ Department of Anesthesiology and Intensive Care Medicine University Hospital S‐H Kiel Germany; ^2^ Department of Neuropediatrics University Hospital S‐H Kiel Germany

**Keywords:** anxiety in children, midazolam, pediatric anesthesia, personality traits, premedication, preoperative anxiety, temperament

## Abstract

**Background:**

Preoperative anxiety is common in young children and may impair cooperation during anesthesia induction. Some temperament traits have been associated with higher anxiety levels in the preoperative phase. While midazolam is widely used for anxiolysis, individual responses vary and may be influenced by underlying psychological characteristics such as temperament.

**Aims:**

This study aimed to examine the association between specific temperament traits and preoperative anxiety in young children and to determine whether these associations persist after midazolam administration.

**Methods:**

This secondary analysis of the ALPAKA trial examined associations between temperament and perioperative anxiety in children aged 2–8 years undergoing elective surgery. Temperament was assessed using the parent‐reported Integrative Child Temperament Inventory (ICTI). Anxiety was rated at two time points before (T1) and after (T2) midazolam administration using the modified Yale Preoperative Anxiety Scale—Short Form (mYPAS‐SF). Additional variables included the Strengths and Difficulties Questionnaire (SDQ) scores, baseline characteristics and prior emotional distress. Spearman correlation (*r*_s_), multivariable and univariate logistic regression analyses were conducted to identify predictors of elevated anxiety (defined as mYPAS‐SF > 30).

**Results:**

Eighty‐nine children were included in the final analysis. Behavioral inhibition was associated with anxiety at both T1 (*r*_s_ = 0.35, 95% CI 0.15–0.53, *p* = 0.001) and T2 (*r*_s_ = 0.46, 95% CI 0.28–0.62, *p* < 0.001). No significant associations were found for other IKT or SDQ subscales. Logistic regression showed that male sex (OR 3.16, 95% CI 1.36–7.19, *p* = 0.011) and prior anesthesia experience (OR 4.28, 95% CI 1.78–10.39, *p* = 0.001) were independently associated with elevated anxiety at T1. A multivariable logistic regression for behavioral inhibition adjusted by sex and prior anesthesia showed for T1 a positive association with elevated anxiety (OR 1.02, 95% CI 1.00–1.05). For T2, the corresponding model showed limited explanatory power.

**Conclusion:**

Behavioral inhibition is a robust predictor of perioperative anxiety in young children, both before and after midazolam administration. Brief screening for inhibition may help identify children at increased risk and guide individualized, risk‐adapted strategies in pediatric anesthesia.

**Trial Registration:**

German Clinical Trial Registration number: DRKS00025411. Principal investigator: Armin Sablewski (15/02/2022, https://drks.de/search/en/trial/DRKS00025411)

## 1 Introduction

Preoperative anxiety in children is a common issue in pediatric anesthesia affecting 40%–70% of patients [1]. Higher levels of anxiety are linked to decreased cooperation during anesthesia induction and a higher risk of postoperative complications such as increased pain and pediatric emergence delirium [2, 3, 4]. Anxiety tends to increase throughout the perioperative period, typically peaking immediately before anesthesia induction [5]. In perioperative settings, children's anxiety is often assessed using observational tools like the modified Yale Preoperative Anxiety Scale (mYPAS) [6, 7].

Midazolam is widely used in pediatric anesthesia to reduce perioperative distress [8, 9]. It has been shown to alleviate anxiety effectively during parental separation and mask induction, without significantly prolonging recovery times [10]. However, its anxiolytic effect varies considerably across individuals. Some children exhibit persistent distress despite adequate plasma concentrations [11], suggesting that psychological or temperamental factors may influence the clinical effectiveness of midazolam [12, 13].

Multiple factors contribute to heightened preoperative anxiety, such as being younger, experiencing parental anxiety, facing language or cultural barriers, socioeconomic status in some countries, and having had negative hospital experiences in the past [14, 15, 16, 17, 18]. Also, personality traits such as child temperament have been identified as an important factor influencing how children respond to stressful situations, including medical procedures [19]. Specific temperament traits—particularly behavioral inhibition, negative emotionality, and low attentional control—have been consistently associated with a higher risk of developing anxiety disorders during childhood [20, 21, 22]. Previous research has examined the role of temperament in perioperative anxiety using instruments such as the Emotionality, Activity, Sociability, and Impulsivity (EASI) inventory [23, 24]. The EASI has contributed important early insights and is based on a biologically oriented temperament model from the 1970s. In contrast, the more recent Integrative Child Temperament Inventory (ICTI) represents an updated, theory‐integrative approach. It captures a broader spectrum of temperament traits and is increasingly used in both clinical and research settings due to its empirical foundation and practical applicability [25].

Given the established links between temperament, anxiety vulnerability, and individual differences in response to anxiolytic medication, it is reasonable to assume that preoperative anxiety may also be influenced by children's underlying temperamental traits. However, few studies have examined this relationship using multidimensional temperament measures.

Therefore, the aim of this study was to examine how pre‐existing temperament traits in children are related to situational perioperative anxiety in young children before and after midazolam administration. We hypothesized that specific temperament dimensions would be associated with increased perioperative anxiety and might also moderate the anxiolytic effectiveness of midazolam. In addition, we explored whether baseline characteristics and prior emotional experiences were associated with elevated levels of preoperative anxiety, aiming to identify simple clinical predictors of distress.

## 2 Methods

### 2.1 Study Design and Setting

This study represents an exploratory post hoc secondary analysis of the ALPAKA randomized controlled trial [26]. Although temperament measures were collected as part of the original study protocol, analyses examining associations between temperament traits and perioperative anxiety were not pre‐registered and were specified after completion of the RCT. The present analysis is therefore exploratory in nature. Key design elements were reported according to the STROBE guidelines for observational studies [27].

### 2.2 Participants

Participants in the original ALPAKA study were children aged 2–10 years scheduled for elective surgery under general anesthesia. For this analysis, only children aged 2–8 years and with complete data on anxiety ratings (mYPAS‐SF) and temperament measures (ICTI) were included in the analyses. Missing data were handled using a complete case analysis.

Exclusion criteria included relevant communication impairments, recent anesthesia exposure (< 6 months), or contraindications to midazolam and/or topical anesthesia (EMLA, local anesthetic cream with lidocaine and prilocaine; Aspen Pharmacare, Durban, South Africa).

### 2.3 Variables and Data Sources

Children's anxiety was assessed using the mYPAS‐SF which is a validated observational tool consisting of 18 items across four domains: activity, emotional expressivity, state of arousal, and vocalization. The total score ranges from 22.9 to 100, with higher scores indicating greater anxiety levels [6].

To assess children's temperament, we used the German version of the Integrative Child Temperament Inventory (Inventar zur integrativen Erfassung des Kind‐Temperaments, IKT), a 30‐item parent‐report questionnaire designed to measure five core dimensions of temperament in children aged 2–8 years: anger/frustration, behavioral inhibition, activity level, attentional persistence, and sensory sensitivity [28]. The ICTI shows good psychometric properties, with an average test–retest reliability of rtt = 0.81 (0.76–0.86), an internal consistency of *α* = 0.79 (0.70–0.85), and evidence of convergent validity (*r* = 0.60–0.82) with established temperament measures such as the Children's Behavior Questionnaire (CBQ) and Emotionality, Activity, Sociability Temperament Survey (EAS) [29]. Each item was rated on a six‐point Likert scale ranging from 1 (never or hardly ever) to 6 (always or nearly always). The ICTI questionnaires were completed by the parents according to the publisher's guidelines. Raw scores were calculated for each subscale and subsequently standardized using age‐ and gender‐specific norms provided in the test manual.

The Parents also completed the German version of the SDQ, a widely used behavioral screening tool for children [30, 31]. The SDQ consists of 25 items rated on a three‐point Likert scale, covering both positive and negative attributes of the child. These items are grouped into five scales: emotional problems, conduct problems, hyperactivity, peer problems, and prosocial scale. A total difficulties score is calculated by summing all subscales except for the prosocial scale. The SDQ can be used for screening, clinical assessment, treatment monitoring, or research purposes and has previously been applied in pediatric anesthesia contexts [32].

**FIGURE 1:**
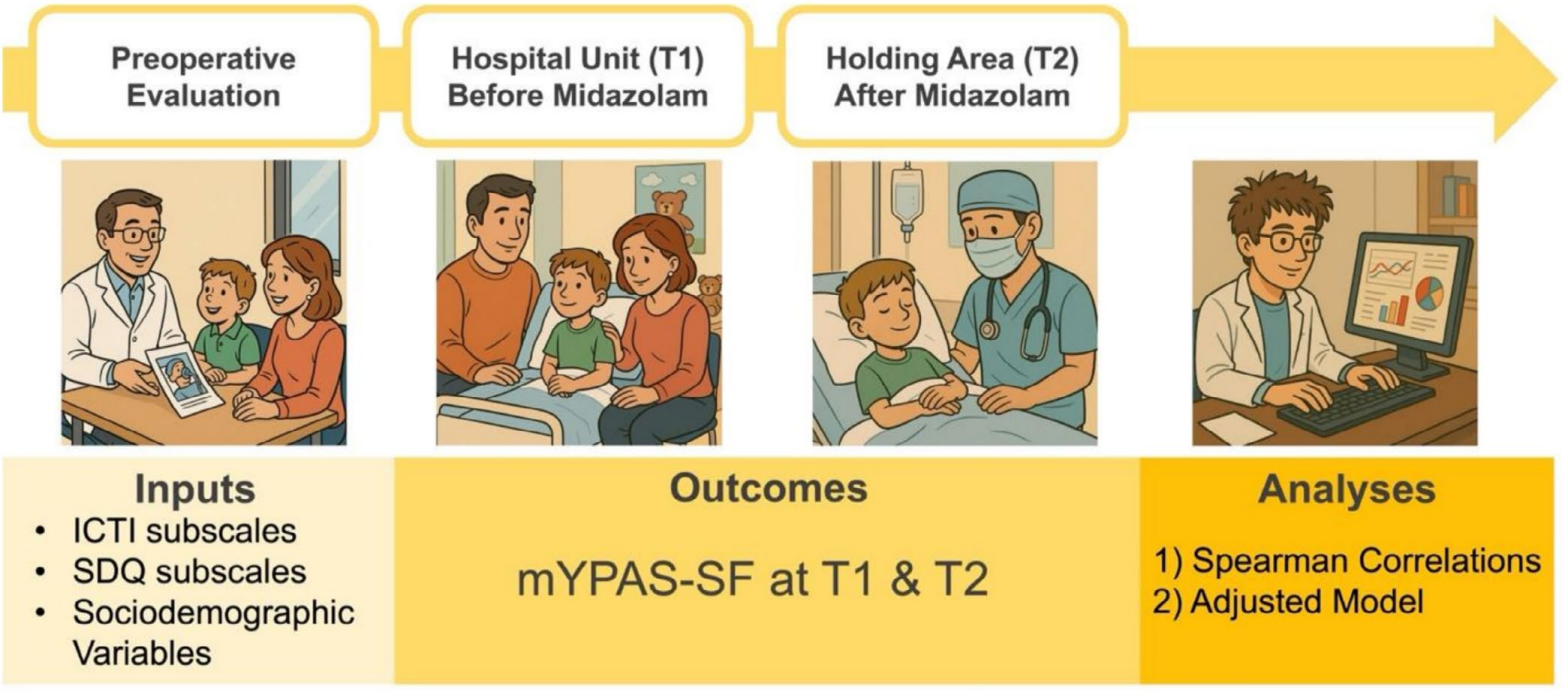
Study timeline, data collection overview and analytical approach. ICTI, Integrative Child Temperament Inventory; mYPAS‐SF, Short Form of the Modified Yale Preoperative Anxiety Scale; SDQ, Strengths and Difficulties Questionnaire; T1, timepoint 1 before midazolam at ward; T2, timepoint 2 after midazolam at holding area.

All participants underwent a standardized protocol: At preoperative consultation, children and their parents were screened for eligibility. After informed consent, patient's baseline characteristics were recorded. Both the ICTI and the SDQ questionnaire were given to the parents during the preoperative evaluation and collected in the recovery room after surgery. On the day of surgery, children's anxiety was assessed at the hospital unit before administration of midazolam, defining timepoint T1. Then, all children received premedication with midazolam (0.5 mg/kg orally or 1 mg/kg rectally ±20%, max. 15 mg) 20–40 min before transfer to the holding area, defining timepoint T2, where full effect of midazolam was expected. Anesthesia was induced with either inhaled sevoflurane or intravenous propofol and maintained according to institutional standards. An overview about the variables and performed measurements is illustrated in Figure 1.

The aim of this analysis was to explore the association between child temperament and preoperative anxiety at two perioperative time points, before and after midazolam premedication. Additional exploratory analyses were conducted for behavioral characteristics assessed using the Strengths and Difficulties Questionnaire (SDQ) and selected clinical and socio‐demographic variables.

### 2.4 Bias

To minimize measurement bias, children's anxiety was assessed by two trained observer/raters who performed all ratings throughout the study. Both raters completed joint standardized training on the mYPAS‐SF prior to data collection and were required to demonstrate consistent and reproducible scoring. Standardized protocols were applied during all assessments to ensure data consistency and integrity. Given the retrospective nature of this subanalysis, the possibility of unmeasured confounding cannot be fully excluded.

### 2.5 Study Size

Sample size for the original trial was determined a priori. No additional power calculation was performed for this secondary analysis. The analysis was based on all available data meeting inclusion criteria for the origin study.

### 2.6 Statistical Methods

#### 2.6.1 Objectives

The objective of this analysis was to examine whether temperament traits, as measured by the ICTI module, were associated with children's preoperative anxiety. The primary endpoint was defined as the Spearman correlation (*r*_s_) between the ICTI subscales and observational anxiety scores assessed with the mYPAS‐SF at two time points T1 (before administration of midazolam) and T2 (after premedication). In addition, correlations between SDQ scores and mYPAS‐SF scores were examined at both time points as well.

#### 2.6.2 Statistical Analysis Plan

Given the multidimensional nature of the temperament measures and the exploratory character of this secondary analysis, a two‐step analytical approach was applied. Exploratory analyses were used to identify relevant temperament dimensions. In a second step, multivariable logistic regression models were fitted to examine whether the temperament dimension identified remained independently associated with elevated anxiety (mYPAS‐SF ≥ 30) after adjustment for age and prior anesthesia experience. To ensure model parsimony and reduce the risk of overfitting given the available sample size, the multivariable models were deliberately restricted to this small set of prespecified covariates. No automated variable selection procedures were applied. Separate models were fitted for T1 and T2.

Model assumptions were assessed by inspection of residuals and linearity of continuous covariates on the logit scale. Multicollinearity was evaluated using variance inflation factors. To assess the robustness of regression coefficients and potential optimism in effect estimates, internal validation was performed using non‐parametric bootstrap resampling with 1000 samples, and bootstrap‐based confidence intervals are reported. No external validation cohort was available. Model fit was assessed using Nagelkerke's *R* ^2^ and the likelihood ratio test comparing the fitted model with the null model.

In an explorative approach, univariate logistic regression analyses were conducted to identify potential predictors of elevated anxiety, defined as a mYPAS‐SF score > 30 as described in a previous study [33]. The following six variables were included as predictors: children's gender, age group (2–5 vs. 6–8 years), only‐child status, presence of emotional stress in the child's history (defined as bereavement or parental separation), living with both parents vs. other (e.g., single‐parent household, patchwork family, or alternating custody model), and prior experience with anesthesia in a medical setting.

#### 2.6.3 Descriptive Statistics and Processing

Categorical variables were presented as absolute and relative frequencies, and continuous variables as medians with interquartile ranges [IQR]. Normality was assessed using the Shapiro–Wilk test. In case of non‐normal distributions, the Wilcoxon rank sum test was used for continuous group comparisons. Categorical variables were compared using Fisher's exact test. All tests were two‐sided, with a significance level of *p* < 0.05.

Correlations between questionnaire scores and mYPAS‐SF scores were analyzed using Spearman correlation (*r*_s_). Statistical analyses were performed using jamovi (version 2.7; The jamovi project) [34], GraphPad Prism (GraphPad Software, La Jolla, CA, USA) and Microsoft Excel (Office 2019, Microsoft Corporation, Redmond, WA, USA). Images were AI‐generated (DALL·E, OpenAI, ChatGPT; Jule 2025 version, USA) without use of any original or copyrighted material.

## 3 Results

### 3.1 Basic Characteristics

One hundred and six children were enrolled in the ALPAKA study. For the present subanalysis, only children aged 2–8 years were considered, resulting in a sample of 92 participants.

Three participants had incomplete IKT data, yielding a final analytic sample of 89 children. Missingness was minimal (3.3% of the eligible sample) and affected only the ICTI; therefore, no imputation was performed. Baseline characteristics of the study population are presented in Table 1.

**TABLE 1:** Characteristics of the children.

Variables	Population, *N* = 89
Age, months	63 [38.5–77.5]
Body weight, kg	19 [14.3–23]
Body height, cm	111 [96–123]
Sex, F:M	48:41
ASA	
1	56 (62.9%)
2	27 (30.3%)
3	5 (5.6%)
Previous illness, yes	41 (46.1%)
Previous anesthesia, yes	48 (53.6%)
mYPAS‐SF T1	33.3 [22.9–43.8]
mYPAS‐SF T2	35.4 [29.2–45.8]

*Note:* Values are presented as median [25th–75th percentile] or number (proportion).

Abbreviations: ASA, American Society of Anesthesiologists Physical Status Classification; F, female; M, male; mYPAS‐SF, Short Form of the Modified Yale Preoperative Anxiety Scale; T1, timepoint 1 before midazolam at ward; T2, timepoint 2 after midazolam at holding area.

### 3.2 Associations Between Temperament Traits and Preoperative Anxiety

#### 3.2.1 Correlation Analyses

Among the ICTI dimensions, behavioral inhibition showed a positive correlation with preoperative anxiety both before (T1: *r*_s_ = 0.35, 95% CI 0.15–0.53, *p* = 0.001) and after (T2: *r*_s_ = 0.46, 95% CI 0.28–0.62, *p* < 0.001) administration of midazolam. No significant associations were found for the other ICTI subscales. Similarly, none of the SDQ subscales significantly correlated with anxiety at either time point. The strongest associations were observed for emotional problems (T2: *r*_s_ = 0.15, *p* = 0.172) and prosocial behavior (T2: *r*_s_ = −0.16, *p* = 0.125). Table 2 summarizes the correlations.

**TABLE 2:** Correlations between IKT and SDQ subscales and observed anxiety (mYPAS‐SF) before (T1) and after (T2) midazolam administration.

	T1	T2
	*r*_s_ (95% CI)	*r*_s_ (95% CI)
ICTI subscale		
Anger/frustration	0.12 (−0.10–0.32)	0.06 (−0.16–0.27)
Behavioral inhibition	0.35 (0.15–0.53)	0.46 (0.28–0.62)
Activity	0.08 (−0.14–0.29)	−0.02 (−0.24–0.19)
Persistence	−0.04 (−0.25–0.17)	0.09 (−0.13–0.30)
Sensory sensitivity	0.08 (−0.14–0.29)	0.13 (−0.08–0.34)
SDQ subscale		
Total difficulties	−0.06 (−0.27–0.16)	0.00 (−0.21–0.22)
Emotional problems	0.01 (−0.21–0.22)	0.15 (−0.07–0.35)
Conduct problems	−0.08 (−0.29–0.14)	0.03 (−0.19–0.24)
Hyperactivity	−0.12 (−0.32–0.10)	−0.14 (−0.35–0.07)
Peer problems	0.01 (−0.20–0.22)	0.10 (−0.11–0.31)
Prosocial behavior	0.04 (−0.18–0.25)	−0.16 (−0.37–0.05)

Abbreviations: CI, confidence interval; ICTI, Integrative Child Temperament Inventory; *r*_s_, Spearman Correlation; SDQ, Strengths and Difficulties Questionnaire; T1, timepoint 1 before midazolam at hospital unit; T2, timepoint 2 after midazolam at holding area.

#### 3.2.2 Multivariable Model: Trait‐Based Predictors of Anxiety

Behavioral inhibition, identified as the only temperament dimension showing a consistent association with anxiety in the correlation analyses, was therefore selected for multivariable modeling and adjusted for age and previous anesthesia experience (Table 3).

**TABLE 3:** Multivariable logistic regression analysis of predictors of elevated preoperative anxiety (mYPAS‐SF ≥ 30) before (T1) and after midazolam administration (T2).

Predictor	T1: OR (95% CI)	*p*	T2: OR (95% CI)	*p*
Inhibition (per unit)	1.02 (1.00–1.05)	0.041	1.02 (1.00–1.05)	0.063
Previous anesthesia yes [reference] vs. no	0.18 (0.06–0.48)	< 0.001	0.56 (0.26–2.41)	0.260
Age (months)	0.99 (0.97–1.02)	0.588	0.99 (0.96–1.01)	0.427

Abbreviations: CI, confidence interval; OR, odds ratio derived from multivariable logistic regression models including inhibition (continuous), age (months), and previous anesthesia experience.

In the multivariable logistic regression model predicting elevated anxiety at T1 (mYPAS‐SF ≥ 30), the model showed a moderate overall fit (Nagelkerke's *R* ^2^ = 0.13) and was significantly better than the null model (likelihood ratio *χ* ^2^(3) = 15.5, *p* = 0.001). Higher behavioral inhibition remained independently associated with elevated anxiety (OR 1.02, 95% CI 1.00–1.05). For T2, the corresponding model showed limited explanatory power (Nagelkerke's *R* ^2^ = 0.05) and did not significantly outperform the null model (likelihood ratio *χ* ^2^(3) = 4.58, *p* = 0.205). Behavioral inhibition showed a similar effect direction but did not reach statistical significance (OR 1.02, 95% CI 1.00–1.05).

### 3.3 Sociodemographic Predictors of Preoperative Anxiety

The multivariable analysis of potential predictors of the presence of preoperative anxiety (mYPAS‐SF ≥ 30) is shown in Table 4. Two variables were significantly associated with increased anxiety before midazolam administration (T1): Male sex (OR = 3.16, 95% CI = 1.36–7.19, *p* = 0.011) and prior anesthesia experience (OR = 4.28, 95% Cl = 1.78–10.39, *p* = 0.001) were associated with a higher likelihood of elevated anxiety. After midazolam administration (T2), none of the examined variables showed a statistically significant association with elevated anxiety levels.

**TABLE 4:** Univariate associations between categorical variables and the child's anxiety before (T1) and after midazolam intake (T2).

Variable	Odds ratio (95% CI)	*p*
Before midazolam (hospital unit, T1)		
Sex (male vs. female)	3.161 (1.36–7.19)	0.011
Age group (2–5 vs. 6–8 years)	0.498 (0.21–1.13)	0.137
Only child (yes vs. no)	1.518 (0.64–3.73)	0.489
Emotional stress history (yes vs. no)	1.111 (0.46–2.78)	0.829
Living with both parents (yes vs. no)	0.612 (0.21–1.64)	0.440
Prior anesthesia experience (yes vs. no)	4.284 (1.78–10.39)	0.001
After midazolam (holding area, T2)		
Sex (male vs. female)	0.869 (0.35–2.24)	0.812
Age group (2–5 vs. 6–8 years)	0.683 (0.27–1.76)	0.476
Only child (yes vs. no)	1.006 (0.39–2.83)	> 0.999
Emotional stress history (yes vs. no)	1.964 (0.71–5.34)	0.210
Living with both parents (yes vs. no)	0.508 (0.18–1.40)	0.244
Prior anesthesia experience (yes vs. no)	1.481 (0.57–3.989)	0.471

Abbreviations: CI, confidence interval; T1, timepoint 1 before midazolam at hospital unit; T2, timepoint 2 after midazolam at holding area.

## 4 Discussion

In this study, we examined trait‐related predictors of preoperative anxiety by relating temperament dimensions and sociodemographic factors to observed anxiety levels before and after midazolam administration. Behavioral inhibition emerged as the only temperament trait consistently associated with higher anxiety levels. Children with higher behavioral inhibition showed elevated anxiety prior to premedication, and although the association persisted at the correlational level after midazolam administration, behavioral inhibition was no longer independently associated with anxiety in adjusted analyses.

Temperament refers to biologically based, early‐emerging individual differences in emotional reactivity and self‐regulation that shape how children respond to their environment [35]. It can be measured using various established instruments. Compared to earlier tools like the EASI, the ICTI offers greater theoretical refinement, age‐ and gender‐specific norms, and has proven feasible in clinical settings due to its brevity and clarity [25].

In the perioperative setting, traits such as behavioral inhibition, negative emotionality, and low activity levels have repeatedly been associated with elevated anxiety [1, 36, 37]. Conversely, sociability and high activity appear to serve as protective factors and our findings align with this pattern, confirming behavioral inhibition as a central risk factor for perioperative anxiety in young children [24].

In contrast, broader psychosocial difficulties assessed by the SDQ were not consistently associated with perioperative anxiety in our sample. This finding suggests that situational anxiety in the perioperative context may be more closely linked to specific temperamental traits such as behavioral inhibition than to general behavioral or emotional problems. In addition, previous research suggests that environmental influences such as parental anxiety, emotional consistency, and previous medical experiences may moderate the relationship between temperament and situational distress [38], but these effects were not pronounced in our sample.

Another question of this study was whether midazolam provides differential benefit depending on individual child characteristics. Behavioral inhibition was the only temperament trait consistently associated with elevated anxiety both before and after midazolam administration, although its independent effect was attenuated after premedication. This pattern suggests that midazolam may reduce observable anxiety while not fully eliminating underlying affective vulnerability in highly inhibited children. The finding that previous anesthesia experience was associated with lower anxiety before, but not after, midazolam administration further supports the notion that pharmacological premedication may override experiential and learning‐related influences on observable anxiety.

In everyday clinical practice, some highly inhibited children may appear quiet and cooperative on the ward, giving the impression that they are coping well. However, despite premedication, they may still show pronounced anxiety at the moment of anesthesia induction. This “hidden vulnerability” makes such children particularly difficult to identify using standard preoperative assessments alone. It may therefore be important to actively identify such profiles and, in clinical practice, adapt sedation strategies accordingly: for example, by considering higher doses or combining pharmacological and non‐pharmacological interventions to more effectively manage perioperative distress.

In this light our findings underscore the relevance of a patient‐oriented approach that accounts for individual psychological profiles. The variability in anxiolytic response observed in our sample reflects previous reports suggesting that midazolam's effectiveness is shaped not only by pharmacokinetic properties but also by underlying psychological and temperamental factors [23]. Notably, midazolam induces a dissociation between explicit and implicit memory in pediatric patients known to exert its primary effects on explicit memory and cognitive awareness, rather than on implicit emotional learning or autonomic stress responses [39]. This distinction may explain why children with high baseline reactivity—such as those with behavioral inhibition—may continue to display observable anxiety despite sedation. Their physiological and affective arousal may persist beneath the drug's amnestic and anxiolytic surface effects, contributing to the variability in observable distress.

For children with pronounced psychological vulnerability, non‐pharmacological measures may therefore play a particularly important complementary role in managing perioperative anxiety. In addition, alternative pharmacological strategies with different neuropsychological profiles, such as alpha‐2 agonists, may warrant consideration in selected cases, as they primarily induce sedation without exerting anxiolytic or amnestic effects [40]. Whether such agents facilitate a more adaptive emotional processing of perioperative stress, or merely attenuate behavioral expression of distress, remains an open question and was not addressed in the present study. This perspective may also help inform the ongoing—and often passionately debated—question of whether midazolam should be used routinely in pediatric anesthesia, by shifting the focus toward risk‐adapted, needs‐based approaches [41]. Furthermore, it may be of interest to investigate under which conditions midazolam could be omitted without causing increased anxiety in children.

Despite its practice‐oriented design, this study has several limitations that warrant careful interpretation of the findings. In methodological terms, this work represents an exploratory post hoc secondary analysis of a previously approved randomized controlled trial and was not pre‐specified in the trial registry. Accordingly, no formal sample size or power calculation was performed for the present research question. The limited sample size restricts statistical precision and increases the risk of Type II error, particularly for null findings. Although a deliberately parsimonious multivariable modeling strategy was applied, several factors known to influence preoperative anxiety in children, including parental anxiety, socioeconomic status, and broader family stressors, could not be incorporated into the multivariable models due to sample size constraints. This omission raises the possibility of residual confounding. As a consequence, some degree of optimism in regression coefficients cannot be excluded, and effect estimates would be expected to shrink in external datasets, as outlined in contemporary prediction modeling frameworks. Overall model fit was modest, precluding strong predictive claims and underscoring the exploratory nature of the analysis. Furthermore, no external validation cohort was available. While internal validation using bootstrap resampling suggested reasonable stability of effect estimates, the generalisability of the findings remains uncertain and requires confirmation in independent samples.

On a clinical and conceptual level, the ICTI is a validated tool for assessing temperament in educational and clinical settings; its use in perioperative contexts is still unusual. Completion requires parental time and engagement. A shorter version and a more pragmatic approach focusing on key traits such as behavioral inhibition may improve its feasibility in routine care, for example by including brief screening questions like “Does your child take a long time to warm up to new situations?” Since all children received midazolam, we could not assess anxiety trajectories in unmedicated children. Finally, data collection occurred during the COVID‐19 pandemic, a period of elevated background stress, which might have affected the generalizability of our results to non‐pandemic settings.

Our findings highlight the value of an individualized approach to managing preoperative anxiety in children, particularly for those with psychological vulnerability. Brief screening for traits such as behavioral inhibition and sensory sensitivity may help clinicians identify children at elevated risk and guide the use of tailored, multimodal interventions. These results support the integration of psychological screening into perioperative risk stratification and underscore the need for prospective studies to evaluate its predictive utility and impact on treatment strategies. Taken together, this study suggests that temperament‐related vulnerability shapes perioperative anxiety expression in a context‐dependent manner and should be considered alongside, rather than replaced by, pharmacological strategies.

## Author Contributions

T.J.: formal analysis, investigation, data collection, resources, data curation, writing – original draft, writing – review and editing. S.W. and T.J.: formal analysis, investigation, data collection, resources, data curation, writing – review and editing. A.P.: conceptualization, formal analysis, writing – original draft, writing – review and editing. T.B. and G.B.: formal analysis, writing – original draft, writing – review and editing. I.L.: conceptualization, formal analysis, writing – original draft, writing – review and editing, project administration. A.S.: conceptualization, methodology, validation, formal analysis, investigation, resources, data curation, visualization, writing – original draft, writing – review and editing, project administration.

## Funding

The authors have nothing to report.

## Ethics Statement

This trial was approved by the local Institutional Review Board (Ethics Committee of Christian‐Albrechts‐University of Kiel, Germany, ethics approval number D418/21) and performed in accordance with the relevant guidelines and regulations as well as in accordance with the Declaration of Helsinki.

## Consent

Informed consent, including consent for publication, was obtained from all parents or legal guardians of participants involved in the study.

## Conflicts of Interest

The authors declare no conflicts of interest.

## Data Availability

The authors confirm that the data supporting the findings of this study are available within the article.
